# Pathogenesis of Hypervirulent Fowl Adenovirus Serotype 4: The Contributions of Viral and Host Factors

**DOI:** 10.3390/v11080741

**Published:** 2019-08-12

**Authors:** Zeng Wang, Jun Zhao

**Affiliations:** College of Animal Science and Veterinary Medicine, Henan Agricultural University, 95 Wenhua Road, Zhengzhou 450002, China

**Keywords:** hypervirulent FAdV-4, hepatitis-hydropericardium syndrome, pathogenesis, viral protein, host factor

## Abstract

Since 2015, severe outbreaks of hepatitis-hydropericardium syndrome (HHS), caused by hypervirulent fowl adenovirus serotype 4 (FAdV-4), have emerged in several provinces in China, posing a great threat to poultry industry. So far, factors contributing to the pathogenesis of hypervirulent FAdV-4 have not been fully uncovered. Elucidation of the pathogenesis of FAdV-4 will facilitate the development of effective FAdV-4 vaccine candidates for the control of HHS and vaccine vector. The interaction between pathogen and host defense system determines the pathogenicity of the pathogen. Therefore, the present review highlights the knowledge of both viral and host factors contributing to the pathogenesis of hypervirulent FAdV-4 strains to facilitate the related further studies.

## 1. Introduction

Fowl adenovirus serotype 4 (FAdV-4), belonging to the FAdV-C species of the *Aviadenovirus* genus, the *Adenoviridae* family, is a non-enveloped, double-stranded DNA virus. Its genome is approximately 45 kb and encodes a number of structural and nonstructural proteins. The FAdV-4 capsid consists of three main exposed structural proteins, hexon, fiber and penton base, with the fiber being noncovalently linked to the penton base. The FAdV-4 capsid is characterized by two separate fiber proteins (fiber-1 and fiber-2) associated with each penton base [1]. FAdV-4 is the primary causative agent of hepatitis-hydropericardium syndrome (HHS), which was first reported in Pakistan in 1987 [2] and subsequent outbreaks have also been reported in Mexico, Russia, Kuwait, Japan, Korea, India, Chile, and South and Central Americas [3,4,5,6,7,8]. HHS mainly affects 3- to 6-week-old broiler chickens [9], and the main clinical symptoms include lethargy, ruffled feathers, prostration and asitia. The typical necropsy lesion is characterized by accumulation of clear, straw-colored fluid in the pericardial sac, as well as multifocal areas of necrosis in liver and hepatitis [8]. Additionally, HHS has been reported in pigeons [10], quails [11], ducks [12], and mandarin ducks [13]. The prevalence of HHS in China was relatively low with only sporadic outbreaks before 2015. However, a dramatic increase in HHS outbreaks began in various broiler-raising provinces of China in May 2015. Accumulating data demonstrated that the HHS in China was caused by a novel genotype FAdV-4, and reproduction experiment with SPF chickens revealed its high pathogenicity [14,15,16,17,18,19,20,21,22]. Given the great threat posed by hypervirulent FAdV-4 strains to poultry industry, elucidating the pathogenesis of FAdV-4 will facilitate the development of effective FAdV-4 vaccine candidates for the control of HHS and vaccine vector. In this review, a comprehensive description of the current understanding in the pathogenesis of FAdV-4 is provided.

## 2. Roles of Viral Factors in the Pathogenesis of Hypervirulent FAdV-4

The genome of FAdV-4 is approximately 45 kb in length and encodes a number of structural and nonstructural proteins. Three main exposed structural proteins, hexon, fiber and penton base, constitute the viral capsid. Hexon contains group-, type- and subtype-specific antigenic determinants, against which antibodies are produced [23]. The fiber protein is related to virus neutralization, cellular receptor binding, tissue tropism and variations in virulence [24]. Each penton unit consists of a penton base anchored in capsid and a projection (fiber) at the distal end [25]. Penton base carries a toxin-like activity that causes cytopathic effect (CPE) on host cells [26].

Owing to the limited available information on the complete genome sequences of FAdV-4 strains with different pathogenicity, the difficulty in manipulating the large size genome of FAdV-4 and a shortage of reverse genetic systems for FAdV-4, the factors determining FAdV-4 pathogenicity profiles remain unclear. Recent studies of fully sequenced genomes of pathogenic and non-pathogenic FAdV-4 isolates implied potential genomic variations associated with virulence [27,28].

### 2.1. Roles of Viral Proteins in the Pathogenesis of Hypervirulent FAdV-4

Sequence alignment revealed that comparing to non-pathogenic strains, hypervirulent FAdV-4 isolated in China have a unique 1966 bp nucleotide deletion on the right region of the genome, resulting in the absence of ORF19, ORF27 and ORF48. Meanwhile, multiple distinct amino acid substitutions were identified on fiber-1, fiber-2, penton and hexon [16]. According to our latest study, a series of chimeric viruses in the background of pathogenic or non-pathogenic FAdV-4 have been constructed and rescued. Mortality patterns showed that seamlessly replacing 1966 bp deletion region of highly pathogenic FAdV-4 with non-pathogenic counterpart did not change the hypervirulent characteristics; however, the pathogenicity of viruses with substituted fiber-2 or hexon gene significantly changed, compared to that of parental strain. These results strongly suggested that the increased virulence of hypervirulent FAdV-4 was independent of 1966 bp deletion, but closely associated with the fiber-2 and hexon genes [27,28].

Previous study suggested that chicken embryo lethal orphan (CELO) virus, belonging to FAdV-1, might bind to the coxsackievirus and adenovirus receptor (CAR) via fiber-1, but initiate the accessory infection through fiber-2 [29], indicating that fiber-2 might be critical for virus-host interaction at early stage. Previous model demonstrated that fiber protein consists of an N-terminal tail region, involved in attachment to the penton base; a C-terminal knob region, which has been assumed to be the ligand for binding to the host receptor; a shaft region, connecting N-terminal tail and knob and its length could influence the interaction between knob and cellular receptor [30]. Many amino acid mutations on fiber-2, such as G219D, P307A, V319I, A380T, conserved in all of the hypervirulent FAdV-4 strains have been identified through sequence analysis, and some of them might be related to the virulence. However, crystal structure of fiber-2 has not been solved yet, so how fiber-2 participates in hypervirulent FAdV-4 infection is unknown, and the spatial positions as well as the effects of mutant amino acids on pathogenicity also need to be further illustrated.

Numerous studies have confirmed that viral proteins can make full use of host factors to facilitate replication and pathogenicity during infection. Research showed that hexon hijacked T-complex polypeptide 1 subunit eta (CCT7) to contribute hypervirulent FAdV-4 replicating in LMH cells, an immortalized chicken liver cell line. Further analysis showed that as a cytosolic chaperone protein, CCT7 might be involved in the folding of hexon protein or play a role in the assembly of FAdV-4 capsid proteins [31]. Additionally, hexon facilitates the infection of Kupffer cells with human adenovirus serotype 5 (HAdV-5) through interacting with scavenger receptor II [32]; penton base is responsible for HAdV-2 internalization through interacting with Bcl-2-associated athanogene 3 [33]. It’s been known that FAdV-4 is a hepatotrophic virus that causes acute and hemorrhagic hepatitis, but the role of hexon in liver injury after hypervirulent FAdV-4 infection and whether penton base participates in virus pathogenicity through influencing cell entry remain unknown.

Apart from structural proteins, the role of nonstructural proteins in virus assembly, stability and pathogenicity cannot be negligible. The late phage nonstructural protein 100K assists chimeric hexon in trimerization to generate recombinant HAdV-5 vectors and improve proliferation efficiency [34]; HAdV-5 100K selectively binds to 5’ noncoding region of viral late mRNA and forms a complex with initiation factor eIF4G and poly(A)-binding protein to promote ribosome shunting and strongly enhance viral protein synthesis [35]; and our unpublished data also shows that hypervirulent FAdV-4 100K promotes virus proliferation in vitro by hijacking members of heat shock protein family. All these data suggest that viral proteins can utilize many kinds of host factors to positively influence virus life cycle. However, further research still needs to look into the specific role of viral proteins in the pathogenesis of hypervirulent FAdV-4.

### 2.2. Immunosuppression Induced by Hypervirulent FAdV-4 May Enhance Its Pathogenicity

Robust innate immune responses can inhibit virus replication and limit viral spread. Conversely, virus has evolved various defense mechanisms to control cell death and repress host antiviral response. The replication cycle of HAdV consists of an early stage and a late stage, and some of early gene products (E1A, E1B, E2, E3, E4) have shown to play important roles in host immunosuppression. HAdV-5 E1A Rb-binding domain markedly repressed NF-κB-dependent defense against TNF-α [36], and E1A CR1 domain inhibited cellular response to interferons [37] and IL-6 gene transcription [38]; E1B-55K participated in countering IFN-inducible genes expression [39] and E1B-19K prevented apoptosis by alleviating p53-mediated transcriptional repression [40]; HAdV-2 E3-19Kgp reduced cell surface expression of histocompatibility class I antigens to escape antiviral cellular reactions [41]. All these data demonstrated that early genes are critical for adenovirus to inhibit host immune response and achieve complete infection.

According to our and others’ research data, hypervirulent FAdV-4 infection causes disintegration of lymphocytes in bursa of Fabricius, severe reduction and necrosis of lymphocytes in the spleen [16], decrease of CD4^+^ and CD8^+^ T-lymphocytes in thymus [42], suggesting that hypervirulent FAdV-4 infection has a profound inhibition effect on immune response. However, the underlying molecular mechanism of hypervirulent FAdV-4 causing immunosuppression is poorly understood, and little is known about whether and which early gene product involves in the process. 

### 2.3. Roles of Mixed Infections in the Pathogenesis of Hypervirulent FAdV-4

Naturally, many chicken flocks will have co-infection with immunosuppressive viruses such as chicken infectious anemia virus (CIAV), Marek’s disease virus (MDV), infectious bursal disease virus (IBDV) and Newcastle disease virus (NDV) [43,44,45]. Immunosuppression resulting from lysis of important cell types may increase the susceptibility to development of more severe diseases. Previous studies indicated that CIAV could assist FAdV-4 in breaking the protection of maternal antibody and cause inclusion body hepatitis-hydropericardium syndrome in layers after using the NDV-attenuated vaccine co-contaminated with FAdV and CIAV. However, no obvious symptoms occurred after using the NDV-attenuated vaccine singly contaminated with FAdV or CIAV [44,45]. 

## 3. Roles of Host Factors in the Pathogenesis of Hypervirulent FAdV-4

Host immune systems have developed multiple ways to recognize viral pathogens. Prompt activation of sensing mechanisms in the host targets each step of infection to detect and eliminate the pathogen. Hypervirulent FAdV-4 infection is initially recognized via pattern recognition receptors (PRRs) [46]. Downstream signaling cascades further elicit the release of pro-inflammatory cytokines into blood to recruit antiviral cells. However, immune response is a double-edged sword, excessive immunoreaction can cause tissue damage and immunopathology [47].

### 3.1. Pattern Recognition Receptors are Crucial to Induce Immune Responses against Hypervirulent FAdV-4

Recent studies have demonstrated that most of the hypervirulent FAdV-4 strains isolated in China are fatal to chickens but not ducks. After infected with HLJDAd15 (duck-isolated strain) [12] or SD0828 (chicken-isolated strain) [46], SPF chickens, but not ducks, exhibited HHS-typical clinical signs and histopathological lesion when exposed at the same doses of virus. By examining the mRNA expression of PRRs, duck retinoic acid-inducible gene I (RIG-I)-like receptors were dramatically upregulated; moreover, virus titers in duck RIG-I transfected chicken embryo fibroblast (CEF) cells were significantly lower than that in empty vector-transfected cells [46]. Previous study showed that in the late stage of infection, adenovirus produces two extraordinarily abundant, highly structured noncoding RNAs termed virus-associated RNAs (VA-I and VA-II) [48], which might form dsRNA-like secondary structures and trigger antiviral immune responses by binding to RIG-I [49]. Phylogenetic analysis showed that RIG-I gene is absent in chickens [50,51], providing a plausible explanation for their higher susceptibility to hypervirulent FAdV-4 virus than ducks. Generally, Toll-like receptors (TLRs) play a critical role in the immune response against DNA virus. For example, TLR9 senses viral genome DNA to produce type I IFN secretion, resulting in antiviral responses; TLR2 and TLR4 recognize some viral-envelop proteins to activate proinflammatory cytokines, leading to inflammatory injury [52]. Previous study supported that HAdV-5 hexon can activate TLR4 signaling pathway and promote NF-κB-dependent transcriptional activation of IL-1β cytokine in the spleen of mice by binding to coagulation factor X (FX) [53]; TLR9 signaling via MyD88 adaptor was required for the production of inflammatory cytokines, chemokines and type I IFN after HAdV-5 administration [54]. However, the avian TLR family is different from the counterpart of mammals [55], and whether TLRs and other type of PRRs in chickens get involved in eliminating the hypervirulent FAdV-4 need to be further illustrated.

In addition, broilers are the dominant hosts for FAdV-4, and once infected, the animals are most likely to develop HHS and other complications. The incidence of HHS and mortality is also higher in broilers than in breeding and laying flocks after infected with FAdV-4 virus [16,56]. So far, no relevant research has been reported to illustrate the underlying mechanism, but the difference in infection time might be the rational assumption. If they were infected at the same age, the outcomes might be similar. As well, surely, the hypothesis needs to be further verified.

### 3.2. Inflammatory Cytokines May Play Roles in the Pathogenesis of Hypervirulent FAdV-4

Cytokines are thought to be important signaling molecules in the induction and regulation of immunoinflammatory responses. Although these molecules play critical roles in eliminating the invading pathogens, exuberant immune responses are positively associated with liver damage [57]. After adenovirus infection, the expression of cytokine genes (including TNF-α, IL-6, IFN-γ, IL-1β, and IL-12) and some chemokine genes (such as IL-8, IP-10, MIP-2, MIP-1α, MIP-1β, and MCP-1) is significantly up-regulated both in vitro and in vivo [58,59]. FAdV-4 can induce liver injury via apoptosis, autophagy, and a severe inflammatory response [59]. Both IL-1β and MIP-2 act as mediators of inflammation in acute liver injury. They are produced by a variety of cell types, such as macrophages, monocytes, epithelial cells, and hepatocytes, in response to viral infection. Down regulation of IL-1β limits acute toxic liver injury in mice [60]. Further research demonstrated that up-regulation of proinflammatory cytokines and chemokines was directly associated with mitogen activated protein kinase (MAPK) signal pathway [61], and inhibition of MAPK cascades decreased the IP-10 chemokine expression as well as liver injury [62]. Recent studies have shown that mRNA expressions of multiple pro-inflammatory and immune cytokines, such as IFN-γ, IL-2 [63], IL-1β, IL-6, IL-8, TNF-α [64,65], IFNs, Mx and OASL [46], increased significantly in the hypervirulent FAdV-4-infected chicken tissues; and our unpublished data also demonstrated that compared with non-pathogenic strains, hypervirulent FAdV-4 infection triggered severe liver inflammation and significantly increased expression level of IL-1β in serum, suggesting that these molecules were positively correlated with tissue damage. Although mRNA expression levels of some anti-viral cytokines, such as Mx and OASL, were highly up-regulated, they might not withstand the “cytokine storms” in the infected organs. This phenomenon indicates that immunomodulatory drugs might be a promising clinical therapy, but how these cytokines, especially IL-1β, get involved in tissue damage after hypervirulent FAdV-4 infection has not been fully understood. Moreover, what role of pathogenicity-associated fiber-2 and hexon play in the boosting expressions of proinflammatory cytokines is worthy of being investigated.

As mentioned above, many chicken flocks often naturally co-infect with immunosuppressive viruses. It is worthy to point out that immunosuppression could actually reduce the effects of a cytokine storm and therefore reduce the mortality associated with these infections. In addition to lysis of important cell types Marek’s disease virus can also interact with inflammatory mediators to change the immune response [66,67]. Thus, the differences in expression patterns of proinflammatory cytokines between the stand-alone infection of FAdV-4 and coinfection of FAdV-4 with other immunosuppressive viruses need to be determined.

### 3.3. Autophagy and Apoptosis May Involve in the Pathogenesis of Hypervirulent FAdV-4

HAdV-5 vector-based vaccines are widely used to prevent infectious diseases, but the main problem is its accumulation in liver [68]. As a hepatotropic virus, hypervirulent FAdV-4 causes liver injury through autophagy and apoptosis. In vivo and in vitro experimental results demonstrated that hepatocyte cells stimulated by hypervirulent FAdV-4 showed nuclear condensation and fragmentation, as well as the increase conversion of LC3-I to LC3-II in a time-dependent manner, indicating that apoptotic and autophagic cell death underlies the mechanism of hepatocytes injury [60]. Research has suggested that oncolytic adenovirus treatment mediated phosphorylation and nuclear translocation of c-Jun N-terminal kinase (JNK) to trigger autophagy [69] and then promote cell lysis and the release of viral progenies at the end of the infectious cycle [70]. As mentioned above, hexon protein plays an important part in promoting HAdV-5 to infect Kupffer cells, so whether JNK related signal pathway is responsible for hypervirulent FAdV-4 induced autophagy and the role of hexon plays in this process still need to be further studied.

Apart from liver injury, hydropericardium is also a typical clinical sign triggered by hypervirulent FAdV-4 infection [16]. After infected with hypervirulent FAdV-4, expression levels of inflammatory cytokines, especially IL-1β, and virus titers in chicken heart increased significantly, and apoptosis of cardiac cells was also observed compared to the uninfected group. However, cardiomyocytes (CM) did not show apoptosis, disorder of myocardial fibers and upregulated expression of inflammatory molecules when directly infected in vitro, indicating that heart might not be the original target of hypervirulent FAdV-4 [64]. However, what deserves to be mentioned here is that the cell types of heart is complex, mainly including cardiac fibroblasts (CF), CM, endothelial cells, and vascular smooth muscle cells [71], and only CM cannot represent the whole heart. Research has already confirmed that CF participates in cardiac inflammation. Under mechanical stress, MAPK pathway in CF was activated and leaded to increased expression of chemokines, which recruit leukocytes to the target tissue to trigger cardiac inflammation [72]. Up to now, it’s hard to isolate and culture different kinds of chicken heart cells and studies about heart damage induced by hypervirulent FAdV-4 are primary and superficial. Whether and how other types of cells in heart are involved in heart injury needs to be further investigated.

### 3.4. Effect of Age on Susceptibility of Chickens to Hypervirulent FAdV-4

Previous studies indicated that the outcome of experimentally induced HHS was dependent on the age of birds [73,74,75]. Most HHS cases were observed in broilers aged from three to five weeks, and the younger ages were associated more severe disease outcomes [76]. One recent study demonstrated that FAdV-4 was pathogenic in geese, and the pathogenicity was also related with age. It was found that the younger the geese were inoculated, the more susceptible they were to the FAdV-4 infection, which indicated that geese had age-related resistance to infection with FAdV-4. Clinical symptoms and pericardial effusion appeared in geese infected with hypervirulent FAdV-4 subcutaneously at 10 days of age, whereas 20- and 30-day-old geese were not susceptible to FAdV-4 [77]. The age-dependent susceptibility to FAdV-4 could be due to the immunodeficiency of young birds, and the underlying mechanisms deserve further studies.

### 3.5. Effect of Maternally Derived Antibodies on the Pathogenesis of Hypervirulent FAdV-4

Vertical transmission is important in the spread of FAdV-4, and consequently, in induction of HHS. A typical outbreak of avian adenovirus is the result of infection occurring in the unvaccinated breeder birds with subsequent vertical transmission to the broilers [78]. As the outbreak progresses through the breeding flock, the number of broilers infected *in ovo* changes and there is a progressive increase in the number of birds with specific maternal antibody. Chicks hatching from infected eggs may excrete virus from the time of hatching, but more typically chicks do not excrete virus until two to four weeks of age. Presumably reactivation of latent virus does not occur until maternal antibody declines [79]. Primarily due to the protection afforded by maternal antibody, HHS is not typically seen in broilers until about three weeks of age, with most field cases occurring between 3 and 5 weeks of age. Those younger than three weeks of age appear to be less susceptible to infection due to maternal antibody. Thus, cases occurring in chickens younger than 3-week-old could be due to a lack of maternal antibody. In view of the significance of both vertical transmission and an early horizontal infection with FAdV-4 for induction of HHS, it is necessary to develop immunization strategies targeting maternal antibody production which can successfully transfer to the progenies.

## 4. Conclusions

The pathogenicity of hypervirulent FAdV-4 is dependent on the function of viral proteins and on host immune responses, implying the important roles of both viral factors and the host immune system in the FAdV-4pathogenesis. Up to now, FAdV-4 pathogenesis-related studies are few and extensive researches urgently need to be carried out. Exploring virulence-related genes, the potential acting mechanism of these genes, and the roles of exacerbated immune response in tissue damage could assist in the development of new strategies to combat hypervirulent FAdV-4 infections.
